# The relationship between gut microbiome and human diseases: mechanisms, predisposing factors and potential intervention

**DOI:** 10.3389/fcimb.2025.1516010

**Published:** 2025-05-06

**Authors:** Mohammad Shabani, Ali Ghoshehy, Amir Mohammad Mottaghi, Zahra Chegini, Azam Kerami, Aref Shariati, Majid Taati Moghadam

**Affiliations:** ^1^ Student Research Committee, Hamadan University of Medical Sciences, Hamadan, Iran; ^2^ Student Research Committee, Tabriz University of Medical Sciences, Tabriz, Iran; ^3^ Student Research Committee, Khomein University of Medical Sciences, Khomein, Iran; ^4^ Department of Microbiology, School of Medicine, Hamadan University of Medical Sciences, Hamadan, Iran; ^5^ Instructor, Department of Nursing, Khomein University of Medical Sciences, Khomein, Iran; ^6^ Infectious Diseases Research Center (IDRC), Arak University of Medical Sciences, Arak, Iran; ^7^ Department of Microbiology, Guilan University of Medical Sciences, Rasht, Iran

**Keywords:** microbiome, gastrointestinal disorders, dysbiosis, metabolic disorders, neurological disorders, and immune-related diseases

## Abstract

The complex interrelation of gut microbiota with human health underlines the profound influence this microbial ecosystem has on mechanisms of disease and wellness. The gut microbiome profoundly impacts various human diseases, encompassing gastrointestinal disorders, metabolic disorders, neurological disorders, and immune-related diseases. Gastrointestinal disorders are closely linked to microbial imbalances in the gut. Metabolic disorders, including obesity and type 2 diabetes, are influenced by the gut microbiota’s role in energy regulation and glucose metabolism. Furthermore, the gut-brain axis highlights the correlation between gut microbiota and neurological conditions such as Alzheimer’s and Parkinson’s. Moreover, the gut microbiome assumes a pivotal function in regulating the immune system, whereby dysbiosis is implicated in developing immunological-related ailments, including allergies and autoimmune disorders. Predisposing factors, including diet, medicines, lifestyle, and environmental influences, are described as having an important role in the composition of the gut microbiome. By understanding these factors, we can get valuable insights into how to intervene to reduce the chances of a disease. Current interventions, including probiotics, prebiotics, fecal microbiota transplants, and lifestyle modification, show promise, but there are still challenges and unanswered questions in this evolving field that may lead to improvements. This review interrelates the complicated gut microbiome with various human diseases, mechanisms, predisposing factors, and potential interventions.

## 1 Introduction

The human gut is the habitat of a complex microbial ecosystem comprised of thousands of species, and its composition varies depending on several factors, including age, diet, socioeconomic status, and geographical location (Serrano-Villar et al., 2016; Sun et al., 2016). The gut microbiota is crucial in the metabolism of nutrients by digesting insoluble fibers, metabolizing nutrients, and producing essential metabolites that impact nutrition. Noteworthy, the gut microbiome is also a significant source of immune interactions, the mechanism of which is currently only partially known. For instance, it was reported that the gut microbiota can enhance the effectiveness of influenza vaccination by stimulating the immune system by releasing flagellin (Dillon et al., 2014; Liu et al., 2017). The gut microbiota has a role in the acquisition of nutrients and the process of energy harvest. It also generates exometabolites, such as short-chain fatty acids (SCFAs), which can potentially influence many metabolic processes in the host (Nicholson et al., 2012; Fernandes et al., 2014).

A diverse array of microorganisms, encompassing bacteria, yeast, and viruses, constitute the gut microbiota. Bacteria are taxonomically categorized based on many organizational units, including phyla, classes, orders, families, genera, and species (Laterza et al., 2016). The primary phyla of gut microbiota are Firmicutes, Bacteroidetes, Proteobacteria, Actinobacteria, Verrucomicrobia, and Fusobacteria. Among these phyla, Firmicutes and Bacteroidetes account for approximately 90% of the gut microbiota species (Arumugam et al., 2011). The composition of gut microbiota exhibits variation based on the anatomical sections of the intestine, which in turn differ in terms of physiological characteristics, pH levels, oxygen tension, substrate availability, and host secretions (Flint et al., 2012). A significant proportion of the gut microbiota comprises stringent anaerobes, surpassing facultative anaerobes and aerobes by up to 100 (Harris et al., 1976). The human gut has been found to harbor about 50 bacterial phyla. However, it is noteworthy that the microbiota is primarily characterized by the dominance of two principal phyla, the Bacteroidetes and the Firmicutes (Schloss and Handelsman, 2004). The quantification of bacterial species within the human gut exhibits considerable variation across different research investigations. However, it is widely acknowledged that people possess a microbial community comprising around 1000 species-level phylotypes (Xu and Gordon, 2003; Claesson et al., 2009; Lozupone et al., 2012).

During the initial year of an individual’s existence, the gut microbiota composition exhibits a comparatively more uncomplicated nature and displays significant interindividual variability (Mackie et al., 1999). Following vaginal delivery, infants possess a microbiota composition that closely resembles that of their mother’s vaginal microbiota. This microbiota mainly consists of dominating groups, namely *Bifidobacterium catenulatum* and *Bifidobacterium longum* (Biasucci et al., 2008; Dominguez-Bello et al., 2010). In contrast, neonates delivered via cesarean section (C-section) are exposed to many bacterial species, including *Staphylococcus* spp., *Propionibacterium* spp., and *Corynebacterium* spp., which originate from both the immediate hospital environment and the maternal skin (Dominguez-Bello et al., 2010; Azad et al., 2013). It is widely accepted that the first colonization of the gut plays a crucial role in determining the composition of the gut microbiota in adults. This observation was substantiated by *Ley* et al., who provided evidence that the gut microbiota of the mice in their study had a strong correlation with that of their mothers, suggesting that kinship plays a significant role in influencing the makeup of the gut microbiota (Ley et al., 2005).

The gut microbiome is a dynamic and diverse community of microorganisms that is one of the cornerstones of human health and is intricately involved in nutrient metabolism, immune function, and energy balance. It is well established that the composition and diversity of gut microbiota are determined by birth mode, diet, and environmental exposures but remain relatively constant at the phylum level. Dysbiosis of the gut microbiota has been related to various gut-related disorders, underscoring its role in maintaining immune homeostasis. Considering the factors involved and mechanisms related to the gut microbiome, we may be able to implement potential interventions to restore or maintain a healthy microbial balance. Understanding these mechanisms and factors opens potential avenues for therapeutic interventions targeting the gut microbiome. Therefore, the current review article will discuss the composition of gut microbiota, its relationship with different human disorders, and various factors that affect the structure of normal gut microbiome.

## 2 Mechanisms of gut microbiome influence on human health

### 2.1 Host-microbiome interactions

The gut microbiome enables a significant expansion of capabilities accessible to the host it colonizes. The genetic makeup and biochemical mixture of the gut microbiota dictate some processes in the gut, which can subsequently influence the maturation and functioning of the immune and nervous systems and metabolic functions. These bidirectional interactions offer a possible mechanistic explanation for how the gut microbiome may modulate systemic metabolic homeostasis (Cox et al., 2022).

#### 2.1.1 Immunomodulation

The majority of the body’s immunity can be attributed to the lymphatic tissue associated with the gastrointestinal tract. It is plausible to conclude that the gut microbiome can significantly influence the homeostasis and development of the adaptive immune system. The gut epithelium serves as a critical anatomical place for the effective interaction among the diverse immune cells and gut microbiome, such as antigen-presenting dendritic cells. Research has demonstrated that this interaction induces gut-resident Foxp3+ regulatory T cells (Tregs), vital cells in modulating immune responses to dietary antigens and gut flora. This process depends on colonizing specific *Clostridia* that produce SCFAs (Ichinohe et al., 2018). Among these SCFAs, butyrate plays a key role as an important factor in maintaining the integrity of CD326+ intestinal epithelial cells and reducing transplant-related diseases in mouse models of allogeneic hematopoietic cell transplantation (Mathewson et al., 2016).

However, the homeostatic maintenance of gut Tregs emerges as required for indigenous *Clostridia* species and a flexible variety of host TCR repertoire. Engineered-transgenic mice expressed a restricted TCRβ repertoire and developed severe colitis associated with a significant reduction in a specific type of Tregs responsible for recognizing gut microbiota and hyperactivation of T helper 17 cells (Th17) (Nishio et al., 2015). These engineered-transgenic mice exhibited no significant changes in the combination of commensal flora, containing segmented filamentous bacteria, which act as an inducer of Th17 cells in the small intestine of mice. Moreover, inflammation in the colon that appeared in these mice was mitigated by “total gut decontamination” using an antibiotic cocktail, offering that the TCR epitopes of effector Th17 cells originate from intestine microbiota more than “self” antigens affiliated with autoimmunity. In this case, it is crucial to highlight that the utilization of broad-spectrum antibiotics, which disrupt anaerobic gut flora, enhances the chance of severe graft-versus-host diseases in the colon following allogeneic hematopoietic cell transplantation in both human and mouse models (Shono et al., 2016).

Reductionist approaches play a significant role in precisely identifying microbial ligands that condition the innate immune response. Many efforts have been made to clarify this issue and understand how different microbes generate distinct innate immune responses. Significant heterogeneity in dendritic cell cytokine responses has been reported in functional phenotypic screening of microbiome isolates from healthy individuals and patients with inflammatory bowel diseases (IBD) (Graham and Xavier, 2023). Unique cytokine profiles in murine myeloid cells result from differential engagement of host Toll-like receptors (TLRs) *in vitro*. *Proteobacteria* and *Bacteroidetes* preferentially induce interleukin (IL)-6, tumor necrosis factor (TNF)-α, and IL-10, while *Proteobacteria* preferentially extract IL-23 and IL-1β. These findings highlight the importance of understanding how specific microbial taxa influence innate immune responses and how dysregulation of these interactions may contribute to IBD and other conditions (Graham and Xavier, 2023).

Initial intestine colonizers are crucial in developing and shaping the primary immune system in infants and neonates. Meanwhile, bisphenol A (BPA) is a chemical commonly found in plastics, such as those used for beverage and food packaging. Perinatal exposure to this substance decreased the frequency of Th1/Th17 cells in the intestinal mucosa of mice. On the other hand, this exposure can lead to other consequences, such as defective secretion of IgA, changes in glucose sensitivity, and decreased abundance of Bifidobacteriales. The significance of these “early colonizers” underscores the need for strategies to address dysbiosis in infant microbiota through personalized functional nutrition (Malaisé et al., 2017; Milani et al., 2017).

Today, due to the importance of the role of commensal and pathogenic microbes in modulating host physiology, the analysis of host cells is used, which has provided new insights. Single-cell RNA sequencing (scRNA-seq) of macrophages from colon tissue of specific pathogen-free (SPF) and germ-free (GF) mice revealed that SPF macrophages exhibited increased expression of genes associated with immune defense, antigen presentation, and oxidative phosphorylation when comparing all clusters of colon macrophages between SPF and GF mice (Kang et al., 2020). Cluster-level analysis showed two specific macrophage clusters significantly increased in SPF mice: 1) CD11c^+^CD206^int^CD121b^+^ macrophages: this cluster exhibited high expression of genes involved in lipid localization, antigen processing and presentation, and cell migration.

2) CD11c^−^CD206^hi^CD121b^−^ macrophages: This cluster showed increased gene expression in wound healing, response to IL-1, vasculature regulation, cytokine production, and apoptotic cell clearance. In contrast, GF mice displayed a high level of macrophage populations with low-level gene expression for stress responses and inflammation. The two distinct macrophage clusters were derived from a common precursor cluster that expressed high levels of CCR2. Increases in pan-macrophage markers and loss of CCR2 were observed in both clusters of macrophages following a pseudo-time course analysis (Kang et al., 2020).

The most recent components of the innate immune system are the innate lymphoid cells (ILCs), which play critical roles in inflammation, mucosal immune regulation, and tissue homeostasis. Based on the differential expression of transcription factors, helper-like ILCs can be classified into three distinct types, each exhibiting unique characteristics. Single-cell RNA sequencing (scRNA-seq) studies have been employed to analyze the response of ILCs to the microbiome and how changes in the microbiome affect ILC biology. Notably, the small intestinal mucosa of mice treated with antibiotics and GFmice exhibited a similar profile of clusters in both groups, significantly differing from those observed in SPF mice. The impact of reducing pre- and post-development microbiota was similarly evident in ILCs (Artis and Spits, 2015; Sonnenberg and Artis, 2015; Gury-BenAri et al., 2016). In GF mice and antibiotic-treated mice, comparisons of the relative abundance of ILC subtypes revealed an expansion of ILC2 and ILC3 cells, accompanied by a reduction in ILC1 phenotypes. Although subpopulations, including ILC1 and ILC2, showed a significant decrease in the expression of ILC2-specific genes, there was an increase in ILC3-specific genes. Furthermore, the expression of the cytokine IL-17a, known to be influenced by the microbiome, was consistently decreased in all subgroups of antibiotic-treated and GF mice (Gury-BenAri et al., 2016).

#### 2.1.2 Metabolic functions

Early evidence suggesting that the intestinal microbiome influences systemic metabolism emerged from various reports. For instance, long-term antibiotic exposure, which has been conducted in humans for over 60 years and remains routine in livestock, has consistently increased body fat mass (Haight and Pierce, 1955; Thaiss and Elinav, 2017). However, a study in mice provided robust descriptions of microbiota alterations associated with obesity. Researchers discovered that feeding genetically obese ob/ob mice the same diet as their lean siblings led to substantial diversity in their microbiome combination. Specifically, the ob/ob mice exhibited a 50 percent decline in *Bacteroidetes* and a corresponding enhancement in *Firmicutes*. In addition, in obese humans and mice, this altered intestinal microbiota demonstrated an independent ability to increase, which, in the context of obesity, was found to have an increased capacity for energy harvesting (Cox et al., 2022).

The intestine microbiome may directly affect energy intake by adjusting the digestion of intricate macronutrients and indirectly by affecting satiety and hunger signals. The microbiome regulates the amount of ingested energy excreted, thereby contributing to energy homeostasis (Turnbaugh et al., 2006; Lund et al., 2020). One mechanism by which the microbiome modulates energy intake is by producing SCFAs, which are byproducts of carbohydrate fermentation through microflora bacteria. These SCFAs may endogenously stimulate the secretion of hunger-regulating peptides, including glucagon-like peptide-1 (GLP-1) and peptide YY (PYY). Following the activation of GIT enteroendocrine cells, these cells specifically express GPR-41 and GPR-43 (G protein-coupled receptors) for SCFAs, leading to the production of satiety-inducing hormones PYY and GLP-1 (Tolhurst et al., 2012; Psichas et al., 2015).

Imidazole propionate, a microbial metabolite derived from histidine, has been identified as a potential connection between the intestine microbiome and type 2 diabetes. The abundance of bacteria responsible for producing imidazole propionate is related to the progress of type 2 diabetes. Imidazole propionate has been shown to inhibit insulin signaling via the AMPK and mTOR pathways, providing a possible description of the microbiome’s effect on a potential target for therapeutic intervention and systemic glucose homeostasis (Koh et al., 2018; Koh et al., 2020). Tryptophan in food is metabolized by the intestinal microbiome, resulting in the production of metabolites such as indole and tryptamine. The aryl hydrocarbon receptor (AHR), a receptor and transcriptional regulator capable of influencing downstream physiological transcriptional programs, can recognize these metabolites. The interaction between SCFAs and AHR is also an intriguing topic, as it represents another potential mechanism of cross-talk between the microbiome’s processing of different macronutrients. As previously mentioned, indole, a tryptophan metabolite, can decrease intestinal permeability (IP) and trigger the release of GLP-1 (Cox et al., 2022). If not regulated by CD4+ T cells, the microbiome’s control of ILC3s affects lipid metabolism through the secretion of IL-22, which modulates the expression of lipid transporters in the epithelium (Mao et al., 2018). This process involves various responses, including the circadian clock protein nuclear factor IL-3-regulated (NFIL3), which regulates lipid absorption in intestinal epithelial cells (Thaiss et al., 2017; Wang et al., 2017a). Additionally, the production of IL-22 in gut ILC3s is partially associate to free fatty acid receptor 2 (FFAR2), which is activated via SCFAs created by microbes (Chun et al., 2019).

#### 2.1.3 Neurological effects

Advancements in medicine have highlighted the microbiome’s role in maintaining homeostasis and regulating main body systems, including the central nervous system (CNS) (Cryan et al., 2020). The strongest evidence for the connection between microbiota and brain function has been observed in GF mice. These mice exhibit abnormal brain development due to the absence of microbiota, which leads to numerous neurobiological changes associated with various neurological disorders. Notable effects include reduced myelination, strain-dependent variations in anxiety-like behavior, and decreased hippocampal volume (Luczynski et al., 2016b; Luczynski et al., 2017). GF mice have demonstrated the microbiota’s involvement in brain signaling, influencing sociability, stress sensitivity, immune function, fear responses, anxiety, and visceral pain. An immature microglial phenotype and significant regulators of neuroinflammation have been identified in GF mice, which struggle to respond effectively to bacterial-related molecular patterns, such as lipopolysaccharides (LPS) (Clarke et al., 2013; Erny et al., 2015; Luczynski et al., 2016a; Luczynski et al., 2017; Stilling et al., 2018).

Furthermore, these mice exhibit enhanced penetration of the blood-brain barrier, compromising its integrity and allowing the translocation of bacterial parts and immune cells into the brain, which in turn affects neuroinflammation. Endocrine and neural routes also influence the secretory products of specialized gut epithelial cells, including Paneth cells, goblet cells, and enteroendocrine cells. These secretions significantly impact the resident microbiota and its survival environment. The gut microbiome modulates CNS activities through various mechanisms: neuronal activation via the microbiome, endocrine signaling through the secretion of 5-hydroxytryptamine by enteroendocrine cells, immune pathways in response to immune cell infiltration and systemic inflammation, and metabolic processes via the production of neuroactive molecules by microbiota. The microbiome influences disease states across a range of neuropsychiatric and neuroimmune disorders and positively affects CNS health through neurodevelopment and cognitive modulation. The gut microbiome and its metabolites can facilitate peripheral immune training when they are sampled by epithelial-attached filamentous bacteria and antigen-presenting cells (Wang and Kasper, 2014; Qiao et al., 2018). Consequently, changes in the microbiome, related to the host’s metabolism, may play a significant role in the progression of neurological disorders, including insomnia, major depressive disorder, autism spectrum disorder, addiction, anxiety disorder, stress, multiple sclerosis, Alzheimer’s disease (AD), stroke, amyotrophic lateral sclerosis, brain injury, Huntington’s disease, Parkinson’s disease (PD), and epilepsy (Moghadam et al., 2023).

### 2.2 Dysbiosis and its implications

#### 2.2.1 Altered microbial composition

Host and microbial communities have evolved strategies to establish a state of equilibrium that benefits both parties. Dysbiosis refers to an imbalance in the abundance of microbial species, which is often associated with the dysfunction of the intestinal barrier and the activation of inflammatory cells. Insufficient regulation of microbial composition (diversity) is likely implicated in the initiation and persistence of numerous diseases, including irritable bowel syndrome (IBS), IBD, diabetes, cancer, and obesity (Belizário and Faintuch, 2018). A potential link between gut dysbiosis and specific human diseases is the abundance of beneficial anti-inflammatory species, such as *Faecalibacterium prausnitzii*, which are more prevalent in healthy individuals compared to pro-inflammatory bacteria like *Bacteroides* and *Ruminococcus gnavus* (associated with IBD). Research has demonstrated that obesity in animal models correlates with a decrease in the Bacteroidetes/Firmicutes ratio and a reduction in the abundance of Bacteroides species. Dysbiosis microbiomes have been observed in various parts of the body, including the microbiomes of the vagina, skin, stomach, and oral cavity, particularly in cases of small intestinal bacterial excessive growth (Belizário and Faintuch, 2018). Factors contributing to dysbiosis in the oral microbiota are implicated in the appearance of periodontal disease, with shifts occurring by diverse mechanisms such as neuro-inflammation triggered by-products of bacteria, accumulation of amyloid beta protein in the brain, damage to oral mucosal barriers, and gut microbiota dysbiosis. These routes enable microorganisms to access the brain via the trigeminal nerve and bloodstream, impacting the nervous system and leading to severe neurological problems, particularly in older patients (Sureda et al., 2020).

#### 2.2.2 Disruption of host-microbiome balance

Changes in gut microbial composition can lead to epigenetic modifications in straight neighbor intestinal cells and distant target cells, such as adipocytes and hepatocytes. Alterations in the intestinal microbiome may be responsible for significant and potentially irreversible consequences on human health, prompting researchers to conduct more targeted investigations to increase innovative plans to safeguard the unification of the microbiota (Moghadam et al., 2023). A fundamental concept frequently highlighted in the medical microbiome literature is “balance.” A well-balanced microbiome is believed to promote health, whereas an imbalance can lead to disease, as disruptions in this equilibrium are associated with various health issues (O’Malley, 2024). Several risk factors have been identified that may contribute to the disarrangement of intestinal microbiome balance. The impact of antibiotics is well-documented, as their use can guide both immediate and long-term shifts in the gut microbiome’s composition. Additionally, significant variation in the intestinal microbiome have been observed in individuals with obesity, as well as those consuming high-fat and high-sugar diets. Environmental influences throughout different life stages are further concepts that play a role in the onset of gut dysbiosis. For instance, variations in microbiome diversity during infancy have been linked to factors such as the type of feeding, method of delivery, and the hospital environment. Furthermore, exposure to xenobiotics, including heavy metals and pesticides, along with social stressors, has been related to the progress of intestine dysbiosis (Capuco et al., 2020).

### 2.3 Molecular mechanisms underlying gut microbiome-related diseases

#### 2.3.1 Inflammation

Dysregulated microbial metabolites, including lipopolysaccharide and ATP, can activate NLRP3 inflammatory vesicles collaboratively. This activation diminishes trans-epithelial resistance and lowers the expression of Zonula Occludens-1(ZO-1), occludin, and claudin-1, resulting in the relocalization of occludin and ZO-1 in Caco-2 cells. Consequently, the integrity of tight junctions in the mechanical obstacle is compromised, leading to increased IP. Additionally, dysbiosis of gut microbiota, in conjunction with pathogenic bacteria and their byproducts, can provoke the intestinal mucosal immune system to secrete TNF-α and IFN-γ (Chu et al., 2023). These cytokines are critical moderators in gut inflammatory diseases, such as IBD. They may affect the function of myosin light chain kinase, occurring in the downregulation and phosphorylation of myosin, along with the redistribution of tight junctions and further junctional proteins. This sequence of events enhances the intestinal epithelial barrier’s transcellular and paracellular penetrance. The increased permeability permits various pro-inflammatory agents to penetrate the submucosa, triggering an inflammatory cascade that disrupts the gut epithelial barrier and fosters chronic mucosal inflammation, characterized by persistent elevations of IFN-γ and TNF-α in the gut mucosa (Chu et al., 2023). The immune system disruption due to intestine microbiota dysbiosis amplifies inflammation in the GIT. Butyrate, produced by commensal gut microbes, effectively promotes the polarization of anti-inflammatory M2 macrophages, positively influencing the immune response and alleviating intestinal inflammation in an animal model of colitis promoted via dextran sulfate sodium. Therefore, SCFAs from gut microbiota may serve as novel activators with anti-inflammatory effects (Haneishi et al., 2023).

Tregs are a specialized subtype of T cells that adjust the immune system, mitigate autoimmune diseases, and preserve tolerance to self-antigens. These cells typically suppress the proliferation and activation of effector T cells. *Clostridium* species are potent inducers of Tregs through butyrate production. In GF mice, a lower concentration of luminal SCFAs corresponds with impaired development of intestinal Treg cells. Thus, a decline in the comparative prevalence of butyrate-producing bacteria, including *Faecalibacterium prausnitzii*, may disrupt mucosal homeostasis.

Furthermore, gut bacteria are necessary to induce effector T cells in the GIT. Th17 cells, a subset of CD4+ T cells, are distinguished by the release of IL-22, IL-21, IL-17F, and IL-17A. Dysregulated Th17 cell activity significantly contributes to developing inflammatory and autoimmune disorders (Andoh and Nishida, 2023).

#### 2.3.2 Epithelial barrier function

The intestinal epithelial barrier, which includes chemical, mechanical, biological, and immune components, prohibits the evasion of toxins and pathogens from the gut lumen (Barbara et al., 2021; Wu et al., 2022). Moreover, the gut microbiota is necessary to support homeostasis within the body, protect the intestinal epithelial barrier, and regulate normal gastrointestinal functions. It significantly impacts the unification and penetration of intestinal epithelial cells by restoring and maintaining tight junctions of mucosal epithelium, thus reducing disruption caused by pathogenic bacteria. The gut microbiota can express genes related to the impact of apoptosis or proliferation of intestinal epithelial cells, tight junction signaling, and aid in restoring a compromised gut obstacle (Monda et al., 2017; Ma et al., 2022). For example, *Lactobacillus royi* LR1 has been demonstrated to mitigate the destruction caused by enterotoxigenic *E. coli* to the membrane obstacle by maintaining the proper localization of ZO-1 and inhibiting its degradation. Additionally, *Lactobacillus royi* boosted the expression of tight junction proteins, thereby strengthening the gut obstacle (Wan et al., 2016; Tulyeu et al., 2019). Therefore, the intestinal biological barrier is important in supporting normal physiological functions. Its unification relies on the effectiveness and stability of chemical, mechanical, and immunological obstacles, the gut microbiota, and the SCFAs it produces. Studies have shown that dysbiosis and reduced microbial diversity in the gut lead to more significant damage to intestinal epithelial cells. This condition is distinguished through a rise in cup cells, a diminish in secretory immunoglobulin A (sIgA) levels—which protect the mucosa—and a reduction in tight junction integrity (Chu et al., 2023). Such changes activate inflammatory vesicles, triggering an immune response in the intestinal mucosa and increasing permeability. This disruption facilitates the immigration of gut pathogens, resulting in systemic or local inflammatory responses. Furthermore, immune-mediated dysfunction of the bowel barrier is thought to play a critical function in the sensitivity to and exacerbation of various inflammatory diseases and autoimmune, including celiac disease, food allergies, IBD, and diabetes (Chu et al., 2023). Dysbiosis typically leads to destruction alterations in intracellular and epithelial cell junctions within the mechanical obstacle, which directly impacts IP. This disruption allows bacteria and damaging substances, such as endotoxins, to arrive in the bloodstream by the gut mucosa, contributing to inflammation. Administering high doses of vancomycin to animal models creates a dysbiosis model characterized by a decrease in Gram-positive microorganisms and an increase in Gram-negative bacteria, primarily from the *Proteobacteria* species (Shin et al., 2015). Intestinal alkaline phosphatase (IAP) plays a role in maintaining intestinal homeostasis by regulating and localizing tight junction proteins. When elevated ATP levels disturb the balance of gut microbiota, many anaerobic archaea exploit the phosphorylation process (Kühn et al., 2020). The mucus layer primarily functions as a chemical obstacle, protecting against pathogens. Immune components within the mucus trap bacteria, preventing their passage into underlying tissues. If the mucus layer is defective or thin, the likelihood of pathogens attacking intestinal epithelial cells increases, leading to a prolonged inflammatory response and potentially triggering IBD. In susceptible individuals, the commensal bacterium *B. thetaiotaomicron* can induce colitis by operating its essential sulfatase activity, which allows bacterial outer membrane vesicles to penetrate the mucus and provoke inflammation (Bersudsky et al., 2014; Sartor and Wu, 2017). If pathogens breach the mucus’s protective barrier and attack the bowel wall’s epithelial cells, the bowel immune system is activated. The first line of defense involves activating senescent goblet cells, which stimulate mucus secretion to help flush out bacteria at the crypt openings. Additionally, antimicrobial peptides released via Paneth cells work with secreted IgA to restrict the survivorship of microorganisms in the small intestine (Chu et al., 2023).

#### 2.3.3 Metabolic pathways

Dysbiosis of the bowel microbiota interrupts the host’s metabolic functions, contributing to the development of inflammatory diseases. The mode of action involved encompasses the biosynthesis of nucleotides and amino acids, disturbances in SCFA production, abnormal catabolism of dietary components, imbalances in redox processes, mucin degradation, sulfur amino acid metabolism, secretory system dysfunction, gene enrichment for pathogenic invasion, and abnormal adhesion (Morgan et al., 2012a). These factors lead to inflammatory diseases associated with impaired bile acid metabolism, compromised digestive functions, and increased hydrogen sulfide production due to gut microbiota dysbiosis. Specifically, dysbiosis negatively impacts SCFA synthesis and the breaking of dietary ingredients, exacerbated by diminished levels of *Clostridium* IV, XIV, *Bifidobacterium*, and *Bacteroides* species (Martens et al., 2018). *Bacteroides*, in particular, contain genes that facilitate carbohydrate metabolism, allowing them to break down indigestible plant and host sugars. As a result, dysbiosis hampers the digestive capabilities of the host bowel, leading to challenges in utilizing and metabolizing dietary elements, which can manifest as symptoms such as malnutrition, indigestion, and a raised possibility of inflammatory diseases (Martens et al., 2011). Gut microbiota primarily adjusts energy absorption via SCFAs that inhibit weight enhancement in rodents and obese individuals. SCFAs interact with GPR, enhancing the expression of GPR41 and GPR43 and upregulating genes correlated to biogenesis of mitochondria such as NRF-1, cyt-c, Tfam, COX IV, β-F1-ATPase, and PGC-1α. Additionally, SCFAs promote beige adipose tissue lipogenesis, increasing free fatty acid oxidation in adipose tissue and triglyceride hydrolysis while mitigating chronic inflammation (Lu et al., 2016). Consequently, decreased SCFAs due to dysbiosis raises the risk of obesity. Research has also shown that excessive SCFAs can contribute to obesity and metabolic disorders. For example, propionate elevates plasma levels of norepinephrine, fatty acid-binding protein 4 (FABP4), and glucagon, stimulating hyperglycemia and glycogenolysis, which can lead to compensatory hyperinsulinemia and insulin resistance (Tirosh et al., 2019). When GPR41 and GPR43 are activated via propionate, it promotes the release of peptide tyrosine-tyrosine that diminishes appetite through stimulating the CNS by the brain-gut-microbe axis (Tolhurst et al., 2012). LPS released via dysbiosis may trigger pro-inflammatory pathways by triggering TLR4 and upregulating NF-κB in adipocytes, initiating pro-inflammatory cascades and releasing inflammatory factors contributing to insulin resistance. Reduced SCFAs inhibit inflammatory responses induced by LPS or TNF-α by a mode of action involving the adjustment of MAPK and NF-κB signaling pathways, serving as essential signaling molecules in the modulation of bowel mucosal immunity. Accordingly, the growth of obesity is nearly linked to bowel microbiome and their metabolites (Chu et al., 2023). Disorder in the metabolism of bile acid diminishes the body’s capacity to fight inflammation and triggers inflammatory illness. The metabolism of bile salts, which is commenced through the bowel microbiome, contains the hydrolysis of bile salts, leading to the formation of preliminary free amino acids and bile acids through the action of the enzyme bile salt hydrolase (BSH) of bacteria. Bacteria that express BSH, mainly from the *Firmicutes, Bacteroidetes*, and *Actinobacteria* phyla, are essential in this metabolic process (Jones et al., 2008; Tian et al., 2020). A dysbiosis distinguished through a reduction in these dominant genera importantly disrupts bile acid metabolism. Bile acids are crucial for breaking down food, aiding in the absorption of cholesterol and fat-soluble vitamins while supporting triglyceride balance and various endocrine functions (Funabashi et al., 2020). They serve as important signaling molecules by activating nuclear receptors containing G protein-coupled bile acid receptor 1, FXR, PXR, and VDR (Wang et al., 2020). This activation prompts the expression of genes involved in maintaining intestinal integrity, suppressing bacterial growth, and preventing mucosal damage, consisting of carbonic anhydrase 12, the pro-inflammatory cytokine IL-18, and inducible NO synthase (Tian et al., 2020). Bile acids also influence glucose and lipid metabolism, energy expenditure, and triglyceride regulation by triggering multiple cellular signaling pathways and nuclear receptors (Chu et al., 2023). For instance, FXR modulates lipoprotein lipase function by promoting the expression of coactivators like Apoc II and suppressing Apoc III (Trauner et al., 2010). The signaling pathways of Wnt/β-catenin, FXR, and NF-κB are interconnected. Research indicated that a lack of FXR in animal models results in enhanced Wnt signaling and primary mortality, which stimulates the generation of TNF-α, macrophages, and neutrophils, contributing to bowel inflammatory diseases (Modica et al., 2008). The mode of action requires dysregulated bowel microbiome releasing LPS that activates NF-κB, guiding to the recruitment of inflammatory cells and increased inflammatory agents. Remarkably, overexpression of NF-κB components p65 and p50 straightly prohibits FXR function, diminishing FXR’s ability to suppress intestinal inflammation and leading to chronic intestinal inflammation (Jia et al., 2019).

## 3 Gut microbiome-associated diseases

**Figure 1:**
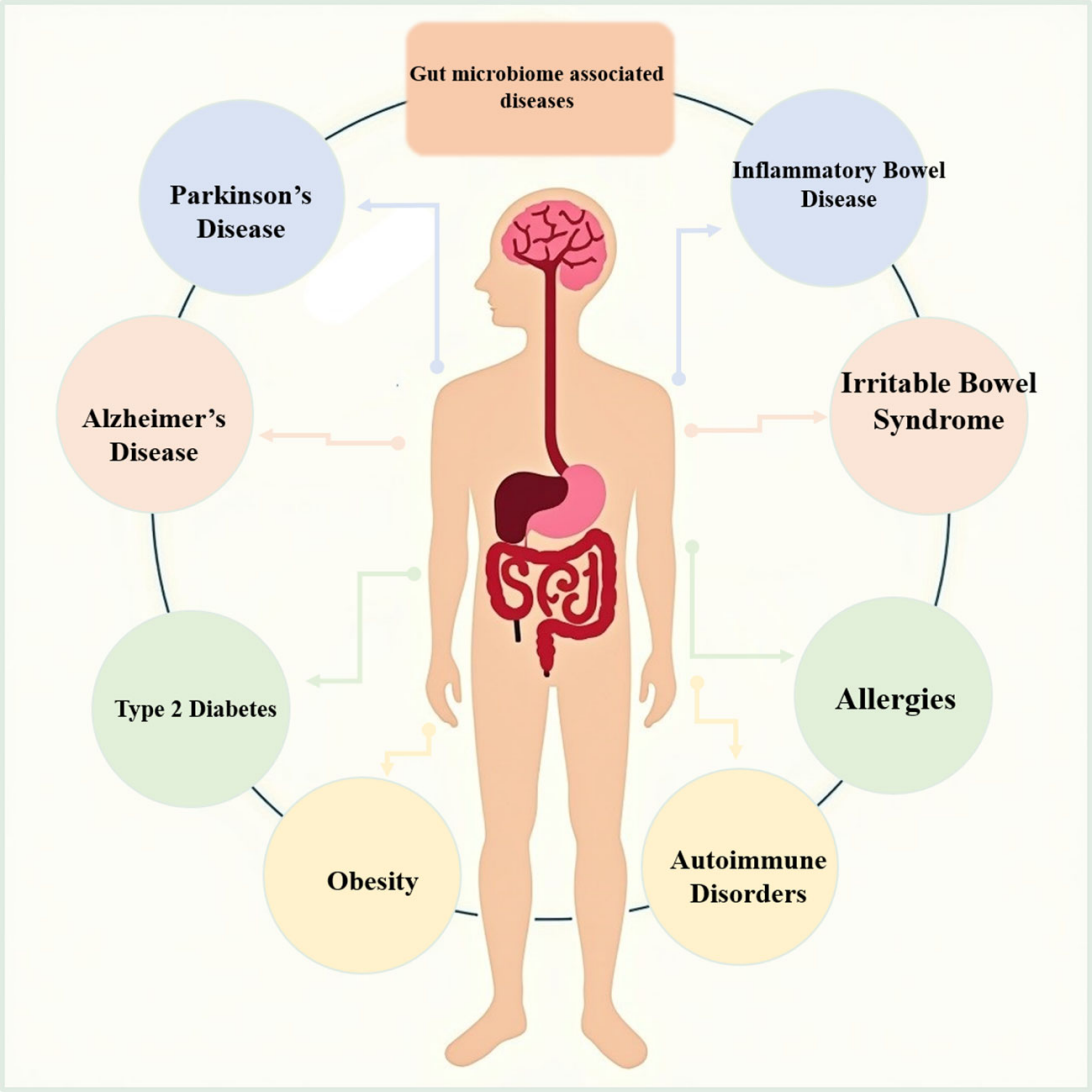
Gut microbiome-associated diseases.

Recent studies have highlighted the gut microbiome’s significant impact on various diseases, including gastrointestinal disorders, metabolic disorders, neurological disorders, and immune-related diseases (**Figure 1**). This section will discuss these diseases and also highlight the effect of these diseases on the microbiome or the effect of the microbiome on these diseases and point out to what extent these two factors can affect each other and to what extent the microbiome can aggravate this disease (**Table 1**).

**Table 1:** Microbiome changes in patients with different disorders.

Study design (year of publication)	Medicine	Illness	Sample source	Sample no	Method	Bacterial diversity	Reference
A metagenomic method has indicated a decrease in the variety of fecal microbiota in individuals with Crohn's disease (2006)	no treatment2: azathioprine 2: 5-ASA2:	CD	Stool	CD:6 HC:6	DNA macroarray-based strategy	InCD: Firmicutes *↓* *Clostridium leptum* phylogenetic group ↓	(Manichanh et al., 2006)
A comparative analysis of the tissue-associated intestinal microflora in individuals diagnosed with Crohn's disease and ulcerative colitis (2006)	All of the patients included in the study were not prescribed any active medications, such as immunomodulators or corticosteroids, except for patient UC6, who received treatment with prednisone and azathioprine	CD UC	Biopsy	CD: 6 UC:5 HC:5	16S rRNA gene sequencing and FISH	The most abundant phylotype overall was *Bacteroides vulgatus*, InCD : *Bacteroidetes*,*Fusobacteria* *Bacteroides fragilis*, *Proteobacteria* ↑ Clostridia, Firmicutes ↓ InUC : *Firmicutes*↑	(Gophna et al., 2006)
This study aims to investigate the genetic risk, dysbiosis, and treatment stratification in inflammatory bowel disease by utilizing the host genome and gut microbiota. (2018)	Not naïve	CD UC	Stool	CD:40 UC:45 HC:146	WGS and shotgun metagenomics	In IBD: Proteobacteria (e.g., *Klebsiella* and *Escherichia* )↑ Bacteroidetes and Firmicutes↓	(Moustafa et al., 2018)
The findings of a pyrosequencing investigation conducted on twins indicate that there are variations in gastrointestinal microbial profiles associated with phenotypes of inflammatory bowel disease (2010)	Antibiotics past 12 months:12 NSAIDS past 12 months:35	UC CCD ICD ICD	Stool	HC:35 UC:16 CCD:12 ICD:15 ICCD:2	Pyrotag sequencing	In ICD: *F. prausnitzii*, *Alistipes* *Faecalibacterium*, *Roseburia* *Ruminococcaceae*↓ Gammaproteobacteria↑ *E.coli*, *Enterobacteriaceae*↑ In CCD: *Ruminococcaceae*, *Faecalibacterium*↑	(Willing et al., 2010)
The investigation of the mucosa-associated microbiota using high-throughput clone libraries provides insights into dysbiosis and distinguishes between inflamed and non-inflamed sections of the intestine in individuals with inflammatory bowel disease. (2011)	_	IBD CD UC	Biopsy	CD:6 UC:6 HC:5	Depth bacterial 16S rRNA gene cloning and sequencing technology	In IBD: Firmicutes↓ *Bacteroidetes*, *Enterobacteriacea*↑ In CD: Enterobacteriaceae, Bacteroidetes↑ Firmicutes↓ In UC: Firmicutes↓ *Bacteroidetes*↑	(Walker et al., 2011)
Impaired functionality of the intestinal microbiota in the context of inflammatory bowel disease and its therapeutic interventions (2012) (2012)	Mesalamine CD67/UC58 Steroids : CD38/UC28 Immunosuppressant: CD47/UC12 Antibiotics : CD15/UC10	CD UC	Stool Biopsy	CD:121 UC:75 HC:27 Indeterminate:8	16S ribosomal RNA (rRNA) gene sequencing	In IBD: Enterobacteriaceae↑ *Roseburia*,Phascolarctobacterium↓ In UC: two types of phylotypes at the genus level, *R oseburia* and *Phascolarctobacterium*, *Leuconostocaceae* ↓ InCD: *Roseburia* and *Phascolarctobacterium*↓ *Ruminococcaceae*, ↓ *Faecalibacterium*↓ *Enterobacteriaceae*↑ In ICD: Roseburia↓ Odoribacter genus, which belongs to the *Porphyromonadaceae* family and to the *Bacteroidetes* phylum↓	(Morgan et al., 2012b)
The changes in the composition and function of the gut microbiome in individuals diagnosed with type 2 diabetes (2020)	Metformin	T2DM	Stool	T2DM:137 HC:179	gene sequencing Metagenomic Sequencing Metabolomic Analysis	DM: *Bacteroidetes*, *Blautia*, *Faecalibacterium*, *Lachnospira*, *Pseudobutyrivibrio*, *Roseburia*, *Prevotella*↓ Proteobacteria, Verrucomicrobia, Actinobacteria *Escherichia*-*Shigella*, *Subdoligranulum*, *Bifidobacterium, Akkermansia*, *Enterococcus*, *Megasphaera*, *Klebsiella*, *Lactobacillus* ↑	(Zhao et al., 2020)
This study examines gut microbiota features in individuals diagnosed with prediabetes and type 2 diabetes. (2021)	Antibiotic: non Probiotic: non	T2DM PreDM	Stool	T2DM:60 PreDM:60 HC:60	16S rRNA gene sequencing, Metabolomic Analysis, Bioinformatics and Statistical Analysis	DM: *Bifidobacteria*, *Bacteroidetes*, Firmicutes (*Lactobacillus*), *Bifidobacterium*, genus *Paraprevotella* from phylum *Bacteroidetes*↓ *Lactobacillus*, *Proteobacteria*, *Prevotella*, *Alloprevotella*, *Megasphaera*↑ PreDM: Proteobacteria, *Megasphaera*↑ Lactobacillus, *Bifidobacterium*, genus *Paraprevotella* from phylum *Bacteroidetes*↓	(Zhang et al., 2021)
The primary bacterial composition of the gut exhibits variations among individuals diagnosed with type 1 and type 2 diabetes, as well as non-diabetic adults (2020)	Not taking antibiotics, corticosteroids, prebiotics or probiotics for the three months preceding the initiation of the study.	T2DM T1DM	Stool Blood samples	T2DM:49 T1DM:21 HC:40	qPCR method	T2DM: *Escherichia, Prevotella, Lactobacillus, Escherichia, Prevotella* and *Lactobacillus* ↑ *Bifidobacterium, Roseburia, Bacteroides, Faecalibacterium*↓ T1DM: *Escherichia, Prevotella, Lactobacillus, Escherichia, Prevotella* and *Lactobacillus*↑ *Bifidobacterium, Roseburia, Bacteroides, Faecalibacterium*↓	(Ejtahed et al., 2020)
Comparative analysis of the gut microbiome in individuals diagnosed with type 2 diabetes belonging to the Chinese minority ethnic groups, including the Uygurs and Kazaks (2017)	Not taking steroids, antibiotics, or probiotics in the last year	T2DM	Stool	T2DM: 20 HC:20	16S ribosomal (r)RNA sequencing technology,	The bacterial families *Lachnospiraceae*, *Ruminococcaceae*, and *Enterobacteriaceae* shown dominance in both healthy individuals and patients with type 2 diabetes mellitus T2DM: Erysipelotrichaceae, (KDM2)↓ *Erysipelotrichaceae*, (UDM2) ↑ *Proteobacteria* (Desulfovibrionaceae Aeromonadaceae),*Veillonellaceae* ↑	(Wang et al., 2017b)
An examination of the gut microbiome makeup in individuals diagnosed with type 2 diabetes residing in northern China (2020)	No previously received pharmacologic treatment	T2DM	Stool	T2DM: 20 HC:40	V4V5 16S rRNA pyrosequencing	T2DM: Firmicutes abundance and a relatively lower abundance of *Bacteroidetes* ↑ genus Dorea (phylum Firmicutes ) *Faecalibacterium prausnitzi* (phylum Firmicutes class Clostridia) genus *Fusobacterium* (phylum *Fusobacteria*) ↑ genus Parabacteroides(Phylum Bacteroidetes), ↓	(Li et al., 2020)
A comparative analysis of microbial community and functions in individuals with type 2 diabetes mellitus and obesity vs a control group of healthy individuals (2020)	-------------	T2DM with obesity	Stool	T2DM:5 HC:6	Metagenomics	T2DM: Bacteroidetes, *B. plebeius*, Proteobacteria↑ Firmicutes, Actinobacteria, probiotic *Lactobacillus*, *Paenibacillus* ↓ *Bacteroides* had the highest prevalence among the genus in the T2DM, accounting for 31.07% of the observed cases. This was followed by *Prevotella*, *Eubacterium*, Rosebiuria, and Faecalibacteriium.	(Wang et al., 2020)
A comparative research was conducted to examine the gut microbiota related with type 2 diabetes in individuals of Han and Mongolian descent. (2021)	Not using antibiotics, corticosteroids and probiotics in the 3 months before registration	T2DM	Stool	T2DM:28 HC:28	16S amplicon sequencing	T2DM : Prevotella, Fastidiosipila Oscillibacter↓ *Alistipes*, *Papillibacter*, Bifidobaicterium ↑	(Li et al., 2021)
This study examines the modification of gut microbiota in individuals recently diagnosed with type 2 diabetes. (2019)	Not taking antidiabetic 3 months after starting treatment	T2DM	Stool	T2DM:50 HC:50	qPCR	T2DM: *Lactobacillus*↑ *C. leptum*, *C. coccoides*↓	(Chen et al., 2019)
This study aims to compare the impact of obesity, xenobiotic intake, and antimicrobial-resistance genes in the human gastrointestinal tract across individuals classified as eutrophic, overweight, and obese (2019)	No documented evidence of intestinal illnesses, diabetes, or the utilization of antimicrobial medications within the preceding 12 months .	Obesity	Stool	Lean (n = 24, BMI 22.2 ± 1.8) Overweight (n = 24, BMI 27.1 ± 1.3) Obese (n = 24, BMI 36.9 ± 6.0)	FISH	Individuals exhibiting obesity demonstrated elevated bacterial density. Individuals with obesity had elevated levels of *Enterococcus*, *Fusobacterium*, and *E. coli* in comparison to lean individuals.	(Sarmiento et al., 2019)
A Comparative Analysis of Gut Microbiota in Lean and Obese Adult Thai Individuals (2018)	Non-administration of antibiotics for at least two months prior to the collection of samples .	Obesity	Stool	Lean (n = 21, BMI 20.7 ± 0.4) Overweight (n = 10, BMI 27.4 ± 0.5) Obese (n = 11, BMI 33.6 ± 1.0)	qPCR	*Staphylococcus, Bacteroidetes, Firmicutes, muciniphila, Akkermansia*, and *Methanobacteria* were lower in individuals with obesity compared with lean. *Ruminoccocus, γProteobacteria, municiphila*, *Christensenella minuta*, and *Akkermansia*, was lower in individuals affected by obesity, compared with overweight. Negative correlation between BMI and *Methanobacteria*, Firmicutes, *Akkermansia, Staphylococcus*, and *municiphila*.	(Jinatham et al., 2018)
This study examines the relationship between the Mediterranean diet, physical activity, and gut microbiome composition in a cross-sectional sample of healthy young Italian adults. (2020)	Not using antibiotics and probiotics in the previous three months	Obesity	Stool	Under weight:7 Normal weight: 106 Over weight: 24 Obese:3	16S rRNA gene sequencing, diet and activity assessments, and statistical analysis statistical Analysis	The *Prevotella* and *Bacteroidetes* had the highest prevalence across all fecal samples. The prevalence of the *Dorea* and Streptococcus was found to be significantly elevated among people classified as overweight or obese. Considerable elevations in the abundance of *Megasphaera*, *Lachnobacterium*, and *Dialister* were seen in individuals classified as overweight/obese and exhibiting low levels of physical activity.	(Gallè et al., 2020)
This study investigates the correlation between body mass index and the Firmicutes/Bacteroidetes ratio within an adult Ukrainian population (2017)		Obesity	Stool	61 individuals Underweight (n = 7, BMI < 18.5) Lean (n = 27, BMI 18.5–24.9) Overweight (n = 16, BMI 25–29.9) Obese (n = 11, BMI ≥ 30)	qPCR	The study findings indicate that individuals who are obese have reduced levels of Bacteroidetes and increased levels of Firmicutes and Firmicutes/Bacteroidetes ratio. There exists a positive link between body mass index (BMI) and concentrations of Firmicutes and Firmicutes/Bacteroidetes ratio, while a negative correlation is observed between BMI and Bacteroidetes.	(Koliada et al., 2017)

ICD, ileal Crohn's disease; CCD, colonic Crohn's disease; HC, healthy; ICCD, ileocolonic Crohn's disease; UC, ulcerative colitis; T2DM, type 2 diabetes mellitus FISH, fluorescence *in situ* hybridization; WGS, Whole genome sequencing; qPCR, Quantitative real-time polymerase chain reaction.

### 3.1 Gastrointestinal disorders

IBD and IBS are prevalent gastrointestinal (GI) illnesses, with prevalence rates ranging from 0.3% to 0.5% and 7 to 21%, respectively, across the global population. Both diseases significantly strain individuals, resulting in a decline in their overall well-being and hindering their capacity to engage in employment and social interactions (Baumgart and Sandborn, 2007; Chey et al., 2015). This section focuses on the intricate interactions of these diseases with the gut microbiome, further underpinned by the role of microbial diversity in the development of diseases and the manifestation of symptoms.

#### 3.1.1 Inflammatory bowel disease

Ulcerative colitis (UC) and Crohn’s disease (CD), often referred to as IBD, are persistent and recurring inflammatory conditions affecting the GIT (Kaser et al., 2010; Khor et al., 2011). The CD is distinguished by a cobblestone-like pattern of inflammation that can manifest at any point along the GIT. Furthermore, this condition is characterized by the presence of ulcerations that can extend throughout the entire intestinal wall, leading to the formation of fissures that have the potential to penetrate the intestinal wall and affect other organs (Nagalingam and Lynch, 2012). The predominant clinical manifestations observed in individuals with CD include exhaustion and abdominal pain. In contrast, in cases of UC, the most frequently encountered symptoms are bloody bowel movements (BM) and diarrhea (Perler et al., 2019). Recently published studies have demonstrated a reduction in the variety of gut microbiota among individuals diagnosed with IBD (Eckburg et al., 2005; Willing et al., 2010; Tong et al., 2013). One of the most commonly observed reductions in intestinal microbial diversity among these patients is the decrease in Firmicutes. Specifically, numerous investigations have documented a decline in *Clostridium leptum* groups, with particular emphasis on *Faecalibacterium prausnitzii* (Manichanh et al., 2006; Wang et al., 2014). The decrease in the population of Firmicutes is of significant importance due to their recognized role as prominent makers of crucial SCFAs, including acetate and butyrate, which are recognized for their robust anti-inflammatory activities (Barcenilla et al., 2000). The observed alteration is correlated with other modifications in the intestinal microbiome specific to individuals with IBD, including an elevation in *Proteobacteria* and *Bacteroides*. Nevertheless, there have been documented instances of a reduction in these bacterial species in other regions (Manichanh et al., 2006; Frank et al., 2007). In contrast to healthy persons, patients with IBD exhibit a concomitant decrease in bacteria possessing anti-inflammatory properties and an elevation in bacteria exhibiting inflammatory capacity (Frank et al., 2007; Peterson et al., 2008; Vich Vila et al., 2018).

#### 3.1.2 Irritable bowel syndrome

IBS is classified as a functional bowel disease (FBD) characterized by the presence of recurring abdominal pain that is linked to defecation or alterations in bowel patterns. Dysfunctional bowel patterns are commonly observed, characterized by constipation, diarrhea, or a combination of both, along with indications of abdominal bloating and distention (Lacy et al., 2016). The precise etiology of IBS remains elusive and is believed to be intricate and influenced by multiple factors. Numerous factors, including genetic predisposition, inflammation, gastrointestinal motor dysfunction, infection, and psychopathological factors, are believed to contribute to the pathogenesis of this condition (Ghoshal et al., 2012). A recent 16S ribosomal RNA-targeted pyrosequencing and machine learning study revealed a distinct gut microbiome pattern associated with severe IBS (Tap et al., 2017). Moreover, individuals diagnosed with IBS may see a decrease in the variety and stability of their gut microbiota (Carroll et al., 2011; Carroll et al., 2012). People diagnosed with IBS may see a reduction in the variety and stability of their gut microbiota. Research findings indicate that persons diagnosed with IBS frequently have diminished microbial diversity and modifications in the composition of their gut microbiota (Bhattarai et al., 2017).

The investigations have documented a decline in helpful bacteria, such as *Bifidobacterium* and *Lactobacillus*, and an elevation in potentially detrimental bacteria, such as *Escherichia coli* and *Clostridium* species, in individuals with this particular disease. Consequently, this disruption in the intestinal microbiota composition can compromise gut integrity (Maukonen et al., 2006; Carroll et al., 2012). Alterations in the makeup of the normal microbiota and disruptions in colonic fermentation have been identified as potential factors contributing to developing symptoms associated with IBS. Notably, a substantial twofold rise in the ratio of Firmicutes to Bacteroidetes has been documented in individuals with IBS (Ponnusamy et al., 2011). Therefore, IBD and IBS are associated with decreased gut microbiota diversity and increased harmful bacteria. IBD patients showed reduced anti-inflammatory bacteria, *such as Faecalibacterium prausnitzii*, while IBS patients showed reduced beneficial bacteria, such as *Bifidobacterium*.

### 3.2 Metabolic disorders

Metabolic disorders like obesity and type 2 diabetes are major global health concerns. In this part, we will concentrate on the involvement of the microbiome in this class of disorders and explore how gut microbes and their respective metabolisms change. More specifically, it is dysbiosis, another term for an imbalance in the gut microbial community, that has been implicated in the development and progression of both conditions.

#### 3.2.1 Obesity

Obesity stands as a prominent contemporary health concern, exhibiting a substantial global surge. There exists a positive correlation between obesity and an elevated susceptibility to Western lifestyle ailments, including type 2 diabetes, cardiovascular diseases, and apnea. Additionally, obesity is linked to an increased risk of mortality (Ogden et al., 2007). Infants born to mothers who experience normal weight growth during pregnancy have elevated levels of *Bifidobacterium* compared to those born to women who experience excessive weight gain. This observation implies that the *Bifidobacterium* group may play a significant role in shaping the microbiota of infants and influencing their weight development (Collado et al., 2008).

Hence, dysbiosis has been implicated in the pathogenesis and advancement of obesity (Turnbaugh et al., 2006). To this end, preliminary evidence suggesting the involvement of gut microbiota in obesity emerged when it was observed that metabolically obese mice harboring a mutation in the leptin gene exhibited a markedly different microbiota compared to animals lacking the mutation (Ley et al., 2005). A subsequent examination revealed that the proportion of Firmicutes to Bacteroidetes in the gastrointestinal microbiota of obese mice shifted towards Firmicutes, while lean mice displayed a predominance of Bacteroidetes (Turnbaugh et al., 2006). This modification in the composition of gut microbes can result in an enhanced ability to extract energy from dietary sources, contributing to weight gain and adipose tissue accumulation (Turnbaugh et al., 2006). Additionally, the impact of the gut microbiota on obesity is mediated by its involvement in regulating appetite and metabolism. Microbial metabolites, such as SCFAs, have the potential to modulate the secretion of hormones such as GLP-1 and PYY, which play a role in the suppression of hunger and the regulation of insulin sensitivity (Canfora et al., 2015).

#### 3.2.2 Type 2 diabetes

Type 2 diabetes is a multifaceted physiological condition that is impacted by a combination of hereditary and environmental factors. It has emerged as a significant global public health issue (Wellen and Hotamisligil, 2005). The findings of a study indicated that individuals diagnosed with type 2 diabetes had minimal divergence from the control group regarding their gut microbiota. However, a decrease in butyrate-producing bacteria, which have the potential to confer metabolic advantages, was detected. Significant elevations in the prevalence of many classifications of opportunistically pathogenic bacteria were also seen, but there was considerable variability in the abundance of these opportunistic pathogen categories (Qin et al., 2012). Therefore, dysbiosis has been observed in diabetic patients, often characterized by a decrease in microbial diversity and a modification in the composition of gut bacteria.

Metabolic endotoxemia, which occurs when bacterial LPS is transported from the gastrointestinal tract to the circulatory system, has been associated with the onset of insulin resistance (Cani et al., 2008). Furthermore, studies have demonstrated that SCFAs generated by gut bacteria via the fermentation of dietary fibers could regulate glucose and lipid metabolism, thus influencing insulin sensitivity and energy homeostasis (Canfora et al., 2015).

Collectively, the development of obesity is accompanied by a shift in the gut microbiota toward Firmicutes and away from Bacteroidetes, coupled with increased capacity for energy harvest and increased weight gain. Indeed, dysbiosis contributes to metabolic syndrome by influencing appetite regulation and inflammation, thereby aggravating metabolic dysfunction. Decreased microbial diversity and increased opportunistic pathogens are characteristics common in type 2 diabetes. These findings support the idea of the critical role of the gut microbiome in driving metabolic disorders, suggesting that targeting this microbial community may improve metabolic health.

### 3.3 Neurological disorders

Recent research has raised suspicions regarding the potential association between dysbiosis of gut microbiota and neurological illnesses, including AD and Parkinson’s disease. The present study examines the microbiome’s contribution to several disorders, focusing on the gut-brain axis and the involvement of microbial metabolites in their pathogenesis. A comprehensive understanding of these mechanisms and interconnections could reveal a novel approach to modulating treatment by targeting the microbiome in various illnesses.

#### 3.3.1 Alzheimer’s disease

Familial AD is an exceedingly uncommon autosomal dominant disorder characterized by its early onset. This condition arises from genetic abnormalities in the amyloid precursor protein and presenilin genes (Blennow et al., 2006). This neurological disorder is described as decreased thinking abilities and memory loss (Zhao and Lukiw, 2015). A study showed how gut microbiota diversity decreases in older and AD patients. This study described gut microbiota’s effects on AD pathogenesis and indicated that loss of microbial diversity could exacerbate cognitive decline (Bostanciklioğlu, 2019). Furthermore, an additional investigation was conducted to explore the correlation between gut microbiota and AD pathology, highlighting the pivotal function of the gut microbiome in advancing the disease (Zhuang et al., 2018).

Gut microbiota may influence the pathogenesis of AD through several mechanisms, including modulation of the immune system, production of metabolites, and direct interactions with the CNS via the gut-brain axis (Zhao and Lukiw, 2015; Minter et al., 2016). One of the critical mechanisms involves the alterations in gut microbiota composition, which have been shown to affect the gut-brain axis, potentially leading to neuroinflammation and the progression of AD (Cryan et al., 2019). The production of microbial metabolites, such as SCFAs, also plays a significant role in AD. SCFAs have been shown to exert anti-inflammatory effects and modulate neuroinflammatory pathways. However, in AD patients, an imbalance in SCFA production has been reported, which may contribute to the disease’s progression (Kobayashi et al., 2017).

#### 3.3.2 Parkinson’s disease

PD is a prevalent and progressing bradykinetic illness that can be effectively clinically identified. The primary established risk factor is advanced age, distinguished by significant pars-compacta nigral-cell depletion and the buildup of aggregated β-synuclein in distinct locations of the brain stem, spinal cord, and cortical areas (Lees et al., 2009). It has been shown gut dysbiosis may lead to increased intestinal permeability (IP) and subsequent neurological dysfunction (Mulak and Bonaz, 2015). Hill‐Burns et al. conducted a comprehensive study on the gut microbiome in PD, supporting the hypothesis that gut dysbiosis is intricately connected to the disease (Hill-Burns et al., 2017).

The gut-brain axis is a bidirectional communication link between the GIT and the CNS, which serves as the primary mechanism by which the gut microbiota influences PD. Empirical research has demonstrated that PD patients frequently display modified gut microbiota composition, impacting gut permeability and resulting in systemic inflammation and neuroinflammation (Mulak and Bonaz, 2015).

It is widely believed that neuroinflammation plays a role in the loss of dopaminergic neurons in the substantia nigra, which is a characteristic feature of PD pathogenesis. Furthermore, the gut microbiota has been associated with the buildup of alpha-synuclein, a protein that induces the formation of toxic aggregates in PD. Numerous studies have indicated that alpha-synuclein aggregates initially manifest inside the gastrointestinal tract’s enteric nervous system (ENS) and spread to the brain through the vagus nerve. This observation implies a potential gut-origin theory for PD (Kim et al., 2019). SCFAs are also known to have a substantial impact on PD. This disease frequently exhibits a decrease in the population of bacteria that produce SCFAs, resulting in diminished levels of these advantageous metabolites and hence contributing to neuroinflammation and neurodegeneration (Unger et al., 2016).

Gut microbiota dysbiosis is also tightly interlinked with PD and is characterized by increased IP and systemic inflammation. The altered gut microbiome plays a role in the gut-brain axis and contributes to the degeneration of dopaminergic neurons. In PD patients, neuroprotective SCFAs are found in reduced amounts, which can further exacerbate neuroinflammation and neurodegeneration, underscoring the role of gut microbiota in PD pathology.

### 3.4 Immune-related diseases

Immune-related diseases, including allergies and autoimmune disorders, have seen a rising prevalence in recent years. This essay investigates how the microbiome in the gut is balanced in these diseases and inquires how the microbiome interacts with the mentioned diseases. Dysbiosis is critical in developing allergic and autoimmune diseases.

#### 3.4.1 Allergies

The prevalence of allergic disorders has emerged as a significant public health concern within prosperous cultures. There is a lack of identification of specific microorganisms that consistently exhibit harmful or allergy-protective properties. Nevertheless, particular investigations have established correlations between diminished colonization rates of *Bifidobacterium* or *Lactobacillus* early in life and subsequent development of allergies (Björkstén et al., 2001; Kalliomäki et al., 2001; Sjögren et al., 2009; Johansson et al., 2011; Hansel et al., 2013). Furthermore, some investigations have provided empirical support for the notion that early colonization by *Clostridium difficile* is a risk factor for subsequent development of allergies (Böttcher et al., 2000; Björkstén et al., 2001; Penders et al., 2007; Van Nimwegen et al., 2011).

Several studies have demonstrated that early-life microbial exposure is critical in shaping the immune system and preventing allergic sensitization. For instance, children born via cesarean section, who are exposed to fewer maternal vaginal and intestinal microbes, have been found to have a higher risk of developing allergies compared to those born vaginally (Dominguez-Bello et al., 2010). Additionally, reduced diversity in the gut microbiome during infancy has been linked to an increased risk of allergic diseases later in life (Arrieta et al., 2014).

The investigation of the gut microbiota in individuals afflicted with atopic eczema revealed that children diagnosed with the disease at one month of age exhibited a notably reduced bacterial diversity, specifically in relation to the Bacteroidetes phylum, in comparison to infants without atopic dermatitis (Abrahamsson et al., 2012). The findings of another study likewise indicated a reduction in the diversity of Bacteroidetes at 12 months of age among individuals with atopic-eczema. These findings suggested that individuals with atopic-eczema may exhibit diminished bacterial diversity compared to health-control subjects. Furthermore, a reduced proliferation of *Proteobacteria*, characterized by lipopolysaccharide molecules in their cell walls, was detected in newborns diagnosed with atopic eczema. LPS possess the capacity to induce an immunological response in the host, and a diminished level of LPS exposure during infancy has been associated with an increased susceptibility to atopic eczema (Gehring et al., 2001).

Noteworthy, the gut microbiota influences immune tolerance through the production of microbial metabolites, which can modulate immune responses and promote the development of Tregs (Furusawa et al., 2013). Tregs are essential for preserving immunological tolerance towards self-antigens and benign environmental antigens, mitigating allergic responses (Atarashi et al., 2013). Therefore, gut microbiota is involved in immune tolerance mediated through microbial metabolites, including SCFAs. This process takes effect in regulatory T-cell induction and prevents allergic reactions.

#### 3.4.2 Autoimmune disorders

Autoimmunity, a clinical phenomenon encompassing seemingly unrelated diseases such as psoriasis, insulin-dependent diabetes mellitus (IDDM), rheumatoid arthritis, multiple sclerosis, and myasthenia gravis, is believed to arise from the malfunction of normal self-tolerance mechanisms, often triggered by environmental stimuli (Himmelweit and Daleeditor, 1956). *Yamamoto* et al. investigated the gut microbiome’s influence on systemic autoimmunity. They summarize evidence showing that gut microbiota plays a crucial role in the integrity of the gut epithelial barrier, which is vital in preventing autoimmune diseases (Yamamoto and Jørgensen, 2020). Another study also highlighted how the gut microbiota collaborates with the immune system to influence the development of autoimmune diseases. This study emphasized that alterations in gut microbiota composition can lead to inflammatory responses, thereby contributing to the pathogenesis of autoimmune diseases (Kosiewicz et al., 2014).

In line with these findings, it was reported that individuals with multiple sclerosis often exhibit a reduced abundance of SCFA-producing bacteria known for their anti-inflammatory properties (Miyake et al., 2015). Similarly, patients with rheumatoid arthritis have been found to harbor increased levels of *Prevotella copri*, a bacterium associated with pro-inflammatory responses (Scher et al., 2013). In systemic lupus erythematosus, alterations in the gut microbiota have been linked to increased IP, or “leaky gut,” allowing for the translocation of microbial products that can trigger systemic immune activation (Zhang et al., 2014). As mentioned, the mechanisms by which the gut microbiome influences autoimmune diseases are multifaceted. Microbial metabolites, such as SCFAs, can modulate immune cell function and differentiation, promoting Tregs and reducing inflammatory responses (Furusawa et al., 2013).

## 4 Predisposing factors influencing gut microbiome composition

**Figure 2:**
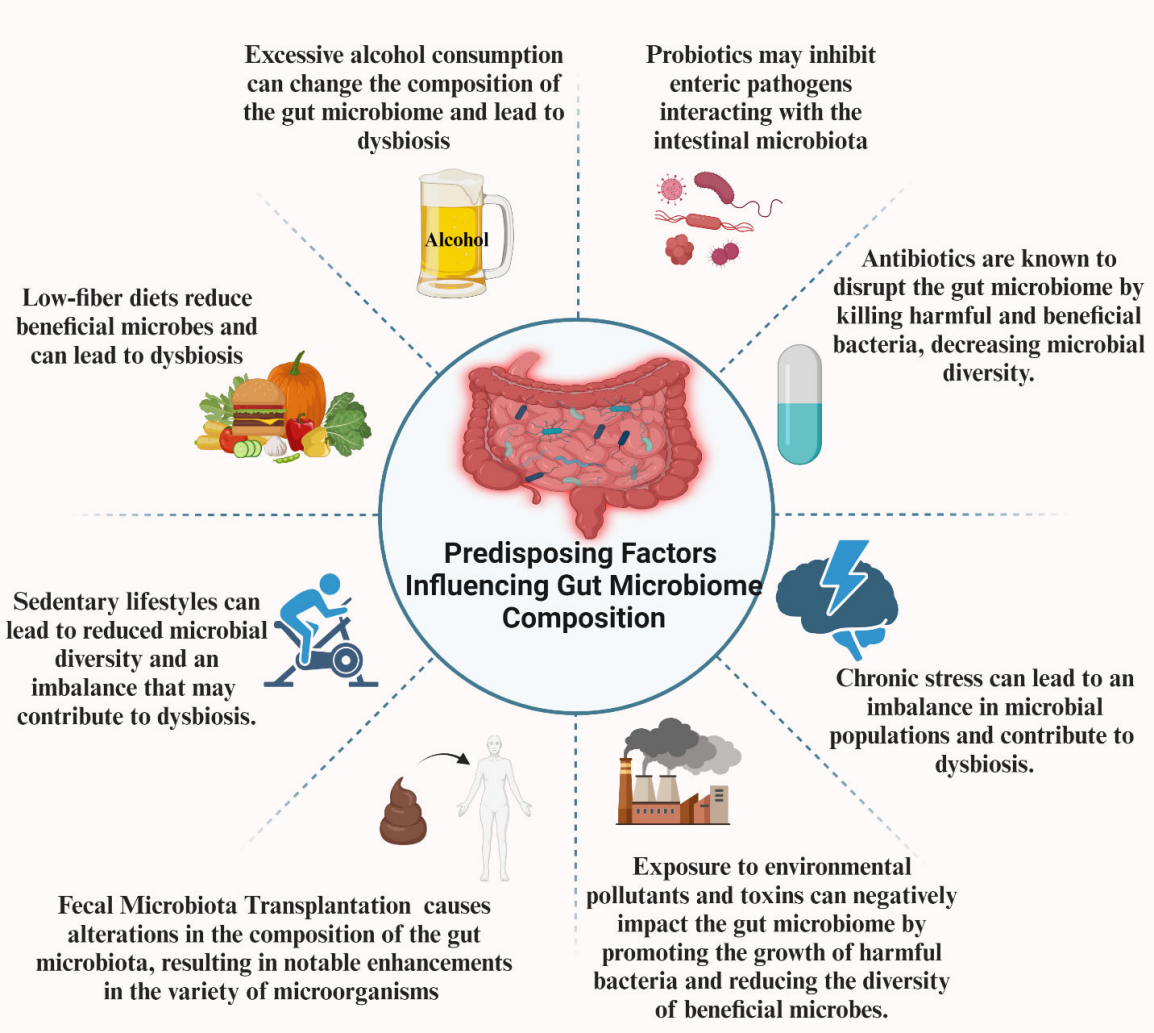
Factors influence the gut microbiota.

As mentioned in the previous parts, the gut microbiome has emerged as a critical player in maintaining overall health and contributing to the pathogenesis of various diseases. However, several factors profoundly affect this microbial ecosystem’s composition and functionality. Diet and nutrition, alcohol consumption, lifestyle habits, antibiotics, and probiotics are among the most significant determinants. Additionally, new therapeutic approaches such as FMT also affect the microbiome. These factors can alter the balance of microbial species, leading to dysbiosis. By understanding how these factors influence the gut microbiota, we can develop targeted strategies to manipulate the microbiome to improve health outcomes and prevent disease (**Figure 2**).

### 4.1 Diet and nutrition

Studies have indicated that diets with low fiber, high protein, and high-fat content can potentially exacerbate intestinal inflammation IP by modifying the composition of microorganisms and metabolites that regulate inflammatory processes (David et al., 2014; O’Keefe et al., 2015; Desai et al., 2016). The diet influences not only the composition of microorganisms but also the regulation of ecological activity and its impact on the host without causing readily observable changes in composition (De Filippis et al., 2016).

It was reported that digestible and non-digestible carbohydrates can enrich *Bifidobacterium* and suppress *Clostridia*. However, it is worth noting that only non-digestible carbohydrates have been observed to further enhance the enrichment of *Lactobacillus*, *Ruminococcus*, *Eubacterium rectale*, and *Roseburia*. To this end, the administration of probiotics and polyphenols augments the growth of *Bifidobacterium* and lactic acid bacteria while concurrently diminishing the presence of Enteropathogenic *Clostridia* species (Singh et al., 2017).

Accumulating evidence implicates the potential association between omega-3 Polyunsaturated fats (PUFAs) and the composition of gut flora. Omega-3 PUFAs can potentially influence the gut microbial ecology, subsequently leading to alterations in the metabolism and absorption of omega-3 PUFAs (Fu et al., 2021). In the adult population, alterations in the gut flora were noted after the administration of omega-3 PUFA supplementation (Costantini et al., 2017). Omega-3 PUFAs have the potential to directly influence the diversity and richness of the gut microbiota. Moreover, omega-3 PUFAs can potentially enhance the population and biomass of advantageous microorganisms, such as *Bifidobacterium* (Kaliannan et al., 2015; Fu et al., 2021). It is widely recognized that the gut microbiota is enhanced by the elevated levels of omega 3 PUFAs, which contribute to a balanced ratio of Firmicutes to Bacteroidetes. This community of bacteria promotes the proliferation of beneficial bacteria belonging to the *Lachnospiraceae* and *Bifidobacteria* families while simultaneously inhibiting the growth of Enterobacteria that produce LPS. Consequently, these factors elicit beneficial effects on the anti-inflammatory properties of the gut microbiota (Costantini et al., 2017).

Previous studies have documented the adverse effects of ingesting added sugars in sugar-sweetened beverages on gut microbiota composition. These effects include an elevation in the Firmicutes/Bacteroidetes ratio and a decrease in the abundance of beneficial butyrate-producing genera, such as *Lachnobacterium* (Ramne et al., 2021). Artificial sweeteners are prevalent in nearly all processed food products, primarily enhancing stability and prolonging shelf life and the overall flavor and texture. Research findings indicated that the consumption of artificial sweeteners has the potential to modify the composition of gut microbiota, leading to undesirable consequences mediated by the microbiota in the host, such as glucose intolerance (Suez et al., 2014; Spencer et al., 2016).

In the end, microbiological enrichment has been linked to diets abundant in fruits, vegetables, and dietary fiber, in contrast to a Western diet that is strong in fat, sweets, and animal protein while lacking in fiber (Cresci and Bawden, 2015). The fecal microbiota of individuals adhering to vegetarian and vegan diets exhibited notably reduced microbial populations of *Bifidobacterium*, *Bacteroides*, *E. coli*, and *Enterobacteriaceae* species, as well as lower stool pH levels in comparison to individuals who consume a combination of both meat and dairy products. In contrast to an omnivorous diet, a vegetarian or vegan diet is characterized by elevated levels of carbohydrates and fiber. This change is attributed to the propensity of the gut microbiota to ferment indigestible polysaccharides into SCFA (Cresci and Bawden, 2015). Upon comparing the Western diet, characterized by its high-fat content, refined carbohydrates, and animal protein, with Eastern diets that primarily consist of carbohydrates derived from plants, rice, vegetables, and fruits, it becomes evident that the microbiota of the Eastern population exhibits a greater prevalence of *Prevotella* spp. as opposed to *Bacteroides* spp. in comparison to the Western population (Ananthakrishnan, 2015; Rajilić-Stojanović et al., 2015). Therefore, a dietary regimen abundant in vegetables and dietary fibers can decrease the pH levels within the intestines, impeding the proliferation of potentially harmful bacteria, including strains of *E. coli* and other members of the *Enterobacteriaceae* family (Tomasello et al., 2014).

### 4.2 Alcohol

The investigations conducted on human and animal subjects have demonstrated that prolonged ethanol use induces dysbiosis, reducing abundances of Bacteroidetes and Firmicutes while enhancing the presence of Actinobacteria and Proteobacteria (Duncan et al., 2004; Mutlu et al., 2012; Tsuruya et al., 2016). The observed alterations in the gut microbiota composition have been associated with the excessive proliferation of intestinal bacteria and compromised permeability, resulting in heightened movement of Gram-negative bacterial products, such as endotoxins, from the intestinal lumen into the systemic circulation (Yan et al., 2011; Mutlu et al., 2012). Furthermore, studies have demonstrated that prolonged exposure to ethanol reduces the prevalence of butyrate-producing bacteria (Dubinkina et al., 2017). Noteworthy, evidence suggests that refraining from alcohol use can effectively repair the integrity of the intestinal barrier in people (Leclercq et al., 2014).

Therefore, alcoholic liver disease is characterized by an aberrant proliferation of bacteria, resulting in a diminished abundance of Bacteroidaceae and probiotic bacteria, specifically Lactobacillus. Etiologies encompass small intestine dysmotility and modifications in the bile acid pool (Kakiyama et al., 2013). Additionally, ethanol disrupts intestinal tight junction integrity (Yin et al., 2012); however, more confirmatory studies are needed in this field.

### 4.3 Lifestyle factors

#### 4.3.1 Physical activity

The results of a study demonstrated that physical activity reinstated bacterial variety in overweight rats (Petriz et al., 2014) and augmented the percentage of Bacteroidetes to Firmicutes, which had been diminished in obesity in mice, in a manner that was directly proportional to the level of exercise intensity (Evans et al., 2014).

In addition, physical exercise was found to enhance the relative abundance of bacteria that produce butyrate and elevate the concentration of butyrate in the intestine (Matsumoto et al., 2008; Evans et al., 2014). According to the findings of *Campbell* et al., it has been proposed that exercised mice harbor microorganisms associated with *Faecalibacterium prausnitzii*. These bacteria have the potential to safeguard the digestive tract by generating butyrate and reducing oxygen levels inside the lumen (Campbell et al., 2016).

In addition to animal studies, human studies also demonstrated that the initiation of exercise during the juvenile period resulted in alterations across multiple phyla, characterized by an upregulation of Bacteroides and a downregulation of Firmicutes. Moreover, while comparing the exercise patterns of juveniles and adults, it was seen that juvenile exercise resulted in a greater range of modifications and an elevation in lean body mass. These findings indicate that participation in physical activity during early childhood can impact the gut microbiota composition (Mika et al., 2015). In line with the mentioned results, significant disparities have been seen between individuals engaged in competitive athletics and those who lead sedentary lifestyles. For example, a higher level of microbial diversity was documented in athletes, which has been strongly linked to their dietary habits and protein intake (Bressa et al., 2017).

Finally, a comprehensive investigation on high-performing rugby athletes revealed that physical training enhanced the variety of gut microorganisms and had a favorable association with protein consumption and creatine kinase concentrations (Clarke et al., 2014). Notably, the Firmicutes phylum exhibited a heightened level of diversity, exemplified by the presence of *Faecalibacterium prausnitzii*, which contributed to preserving a more favorable intestinal environment (Clarke et al., 2014). Furthermore, physically active people had a microbiome characterized by a higher abundance of taxa that produce butyrate, including *Clostridiales*, *Roseburia*, *Lachnospiraceae*, and *Erysipelotrichaceae*. This bacterial community led to an augmentation in butyrate production, a sign of healthy gut function (Estaki et al., 2016).

Studies on the gut microbiome are limited and are fewer than research regarding diet modifications; however, the impact of physical activity on the gut microbiome is considered significant but not enough, and more research in the area is needed. The research indicates in animal studies that exercise restores bacterial diversity within the microbiome and changes the microbial populations within the gut. In human studies, physical activity enhanced bacteria diversity, increased specific butyrate-producing bacteria, and restored gut health. The research also points out that physical activity in early life has more of an impact on the growth of the gut microbiome than physical activity during adulthood, suggesting that physical activity could play a role in controlling gut health and general health.

#### 4.3.2 Stress

Previous studies have demonstrated a significant correlation between severe physical and psychological stress and changes in the makeup of the intestinal microbiota, as well as an increase in IP (van Wijck et al., 2012; Mayer et al., 2015; Clark and Mach, 2016; Dokladny et al., 2016). Nevertheless, the existing knowledge regarding the involvement of the intestinal microbiota in facilitating the impact of physical, psychological, and environmental stressors on the intestinal barrier predominantly relies on data obtained from animal models, which may not comprehensively reflect the human biological context (Mayer et al., 2015; Clark and Mach, 2016). A Recently published study reported a correlation between gastrointestinal distress experienced during military training and elevated levels of stress, anxiety, and inflammation, as well as enhanced permeability of the intestinal and blood-brain barriers (Li et al., 2013; Li et al., 2014).

The intestinal barrier integrity is negatively impacted by a multiple-stressor environment that includes high levels of physical activity, poor calorie intake, muscle injury, and inflammation. Changes in the composition and metabolism of the intestinal microbiota accompany this unfavorable effect. The observed correlations among elevated IP, the pre-STRESS microbiota, and stool metabolites linked to the microbiota indicate that directing efforts toward the intestinal microbiota may offer innovative approaches to preserving intestinal barrier integrity in physiological stress (Karl et al., 2017).

Hence, the intestinal microbiota could serve as a mediator of immune response to severe physiological stress, suggesting that addressing the microbiota before stress exposure could be a viable approach for preserving immune function. One potential strategy to mitigate the observed connections between changes in IP during stress is to enhance microbiota diversity and Actinobacteria relative abundance while simultaneously reducing Proteobacteria relative abundances before stress exposure. A higher level of microbiota diversity is commonly seen as a reliable indicator of a robust intestinal ecology, as it has been consistently linked to a reduced risk of chronic diseases (Claesson et al., 2012; Huttenhower et al., 2014). Similarly, certain species within the Actinobacteria phylum, such as those classified under the *Bifidobacterium* and *Collinsella* genera, have advantageous anti-inflammatory and immunomodulatory properties that have the potential to safeguard the integrity of the intestinal barrier in times of stress (Ohland and MacNaughton, 2010; Andrade et al., 2015).

Therefore, as mentioned, the gastrointestinal microbiome exhibits sensitivity to stress and its associated mediators. Enteric bacteria exhibit a direct response to the host’s production of stress-related neurochemical mediators, exerting an influence on the host’s response to a bacterial infection (Lyte et al., 2011). High-intensity exercise is a physiological stressor that has the potential to induce gastrointestinal disorders. It was reported that a significant proportion, ranging from 30% to 90%, of distance runners have encountered gastrointestinal issues associated with physical activity (de Oliveira et al., 2014). In contrast to the advantages related to consistent physical activity, prolonged and excessive exercise has the potential to adversely impact intestinal function. Interstitial ischemia may occur as a consequence of prolonged intestinal hypo-perfusion caused by high-intensity exercise. It is noteworthy to mentioned that elevated IP can lead to heightened vulnerability of the gut to endotoxin translocation (Brock-Utne et al., 1988; Oktedalen et al., 1992; Pals et al., 1997).

*Yu* et al. reported a significant drop in the relative abundances of Bacteroidetes and Firmicutes in rats exhibiting depressive-like characteristics. Furthermore, experiments conducted on mice subjected to prolonged stress revealed decreases in the abundance of the *Bacteroides* genus (Bailey et al., 2011; Yu et al., 2017). Previous studies have demonstrated that mice experiencing stress have higher levels of the genus *Clostridium*, consistently aligning with the gut microbiota composition observed in rats that have been separated from their mothers (Bailey et al., 2011; O’Mahony et al., 2011; De Palma et al., 2015). It is widely recognized that antidepressants possess antibacterial properties, which enable them to influence the pathophysiology of anxiety and depression by altering both brain biochemistry and the composition of the gut microbiota (Munoz-Bellido et al., 2000; Lieb, 2004). In contrast, it has been observed that some antibiotics, namely β-lactams and tetracyclines, exhibit promising antidepressant characteristics in both rodents and humans (Miyaoka et al., 2012; Mello et al., 2013). Finally, evidence from epidemiological research has indicated a potential association between some kinds of antibiotics, such as fluoroquinolones, and the onset of depression and anxiety (Rollof and Vinge, 1993; Grassi et al., 2001; Ahmed et al., 2011; Kaur et al., 2016).

### 4.4 Medications

#### 4.4.1 Antibiotics

Antibiotics can target pathogenic microorganisms and the microbial populations associated with the host species in the gastrointestinal tract. Most antibiotics have broad-spectrum activity, enabling their application in treating numerous illnesses. Hence, while antibiotics are specifically formulated to target pathogenic microorganisms, they also impact associated microbiota, resulting in a persistent adverse influence on the gut microbial population even after the cessation of antibiotic treatment (Jernberg et al., 2007). A reduction in the microbiome variety is commonly observed after antibiotic therapy. While most of the microbiota reverts to their pretreatment levels, certain compositions are permanently eliminated from the community (Löfmark et al., 2006). The spectrum of antibiotic activity significantly impacts the alteration of gut microbiota composition. The antibiotic dosage also plays a crucial role in defining its ecological influence on the microbiota. It is important to highlight that the impacts of antibiotic administration extend beyond oral administration. The use of antibiotics by intravenous routes can potentially impact the composition of the gut microbiota, as these medicines are assimilated into bile and subsequently released into the intestine through the biling system (Smith et al., 2002).

Alterations in gastrointestinal immunity heighten the vulnerability of the host to pathogenic infections. For example, the administration of metronidazole, an antibiotic specifically designed to combat anaerobic bacteria, leads to a decrease in the structural integrity of the mucus layer and an increase in the rate of mucosal adhesion by *Citrobacter rodentium* (Wlodarska et al., 2011). The alteration of gut microbiota composition and functionality after antibiotic therapy engenders a metabolic environment that promotes the proliferation and establishment of *C. difficile* infection (CDI), leading to infectious diarrhea (Theriot et al., 2014). Although antibiotic medication is considered the primary risk factor for colonization by *C. difficile*, the recommended treatment for CDI is continued antibiotic therapy. The repetitive administration of antibiotics is frequently required to manage recurring CDI, resulting in additional disturbance to the equilibrium of the microbiota (Chang et al., 2008; Hamilton et al., 2012).

Studies have revealed a significant association between the administration of antibiotics in the initial year of life and the subsequent onset of asthma between the sixth and seventh years of life (Kozyrskyj et al., 2007; Risnes et al., 2011). The initial application of macrolides in Finnish children resulted in the developing of a unique microbial composition characterized by a decrease in Actinobacteria and an increase in Bacteroidetes and Proteobacteria. Additionally, an observed rise in the expression of antibiotic-resistance genes was observed. The above profile exhibited a favorable correlation with either a subsequent onset of asthma or an elevation in body mass index (Korpela et al., 2016).

Research studies have demonstrated that the administration of various antibiotics, such as penicillin, ampicillin, and cephalexin, to preterm newborns leads to an elevation in the proportion of potentially dangerous *Enterobacteriaceae*, while concurrently reducing the proportion of microbial taxa associated with a healthy microbiome (Tanaka et al., 2009; Greenwood et al., 2014; Arboleya et al., 2015). There exists a correlation between antibiotic medication in newborns and an elevated susceptibility to necrotizing enterocolitis, which is recognized as the primary cause of morbidity among infants (Lin et al., 2008). Hence, a complex relationship exists between antibiotics and the gut microbiota. Antibiotics cause disturbances to the indigenous microbiota, resulting in the elimination of pathogenic bacteria and potentially disrupting the natural microbiota. Consequently, this disruption can develop infectious diseases and various gastrointestinal complications. Furthermore, the gut microbiota possesses the capacity to alter certain medications throughout the process of metabolism. Therefore, the metabolic byproducts of these pharmaceutical substances can potentially disrupt the typical microbiota makeup, resulting in significant adverse effects (Anwar et al., 2021).

#### 4.4.2 Probiotics and prebiotics

Probiotics, which are living microorganisms believed to impart health advantages when administered appropriately, possess the potential for targeted therapy (Hill et al., 2014). Prebiotics are a class of chemicals that undergo selective digestion to promote the growth or proliferation of helpful microorganisms, hence offering significant potential for the customization of therapeutic interventions (Gibson et al., 2017).

Previous studies have demonstrated that the administration of probiotics, specifically *Bifidobacterium infantis* and *Lactobacillus acidophilus*, to infants with extremely low birth weight has resulted in a drop in morbidity rates, an increase in daily weight gain, and a reduction in hospital stay durations (Abrahamsson et al., 2014; Härtel et al., 2014). Different mechanisms have been proposed to elucidate the beneficial effects of probiotics. Probiotics have been shown to possess the ability to impede the invasion of mucosal cells, enhance the integrity of the intestinal mechanical barrier, diminish the translocation of microorganisms, restore the immunological function of the mucosal lining, and mitigate local inflammation while reducing systemic inflammation (Kim et al., 2016; Zilberman-Schapira et al., 2016; D’Angelo et al., 2017; Nair et al., 2017). Additionally, probiotics have the potential to possess inhibitory effects on enteric pathogens, engage in interactions with the intestinal microbiota, and have modulatory effects on the immune system either directly or via altering the composition of the gut microbiota. *Bifidobacterium* and *Lactobacillus* species are the predominant probiotics employed in food applications (Salazar et al., 2017).

In a randomized placebo-controlled trial, a cohort of 60 overweight healthy adults were administered probiotics comprising three strains of *Bifidobacteria*, four strains of *Lactobacilli*, and one strain of *Streptococcus*. The study reported notable elevations in total anaerobes, aerobes, *Bifidobacteria*, *Lactobacillus*, and *Streptococcus* compared to the placebo administration. Furthermore, the participants exhibited decreased total coliforms and *E. coli* levels and reduced high-sensitivity C-reactive protein, LDL-cholesterol, triglycerides, total cholesterol, and VLDL-cholesterol. In line with these results, probiotic-enriched yogurt has demonstrated a notable capacity to substantially decrease the levels of the enteropathogenic microorganisms *E.coli* and *Helicobacter pylori* (Liu et al., 2010; Yang and Sheu, 2012).

#### 4.4.3 Fecal microbiota transplantation

Microbiota-based therapies, including FMT, have garnered increasing attention as innovative therapeutic strategies. FMT is a method that entails the complete transfer of feces from one person to another to restore the gut with a “healthy” microbiota (Vindigni and Surawicz, 2017). FMT has expanded the range of therapeutic alternatives accessible to those afflicted with ailments linked to disruptions in the gut microbiome. FMT has been given for various disorders and has demonstrated efficacy in alleviating diverse patient symptoms (Kang et al., 2017). The mechanisms involved in FMT treatment are likely to include the introduction of bacteria from healthy donors to restore the balance of the gut microbiome, leading to improved intestinal barriers, reduced mucosal inflammation, and regulated interactions between innate and adaptive immune cells to suppress autoimmune disorders (Yang et al., 2023).

The findings of a recent meta-analysis indicated that FMT exhibited superiority over placebo in producing combined clinical and endoscopic remission in patients with UC. Furthermore, compared to existing targeted pharmacotherapies, FMT demonstrated similar efficacy in promoting clinical remission, clinical response, and endoscopic remission (El Hage Chehade et al., 2023). Another study also assessed the characteristics and results of individuals with UC who underwent FMT for recurrent CDI. In the group of patients diagnosed with UC, FMT showed a high efficacy level in treating recurrent CDI without any significant adverse effects. Furthermore, most patients reported remission or improved UC activity following FMT (Haifer et al., 2022).

However, several factors have posed challenges to the utilization of FMT in clinical settings. These factors include the identification of a suitable donor, the influence of donor-specific microbiota dynamics on host-related factors, the requirement for specific techniques, the need for specialized experimental conditions, and the necessity for professional evaluation and implementation by specialized individuals. Additionally, the lack of satisfactory clinical evidence and the variability in research outcomes have further hindered the application of FMT in clinical settings (DeFilipp et al., 2019). Apart from the risk factors mentioned above, FMT has the potential to induce chronic and non-communicable diseases, including cancer, metabolic problems, and inflammatory diseases (Ianiro et al., 2022).

### 4.5 Environmental factors

#### 4.5.1 Pollution

Particulate matter and ozone, both constituents of air pollution, are currently recognized as having significant health implications. Specifically, ozone and particulate matter have been found to facilitate increased gut permeability and may disrupt the integrity of tight junctions within intestinal cell walls (Bhalla, 1999). Although extensive research has been conducted on the effects of environmental contaminants on many health issues, there remains a dearth of knowledge regarding the influence of air pollution on the gut microbiome. The study conducted by Kish et al. revealed that the consumption of pollution particles through chow resulted in notable alterations in the composition of gut microbiota, namely in the relative abundances of Bacteroidetes, Firmicutes, and Verrucomicrobia (Kish et al., 2013).

According to the findings of the Center for Disease Control and Prevention (CDC), it has been established that there is no recognized safe blood level for Pb (Betts, 2012). In a separate investigation, adult C57Bl/6 female mice were subjected to a drinking water composition containing 10 ppm PbCl2 for 13 weeks. The concentration of PbCl_2_ used in this study was 2 mg/kg body weight/day. The findings of this study indicated that the presence of this chemical had an impact on the trajectory of the gut microbiome and the diversity of its phylogenetic relationships. Additionally, alterations in gut metabolism were observed when fecal samples were evaluated at 4 and 13 weeks after exposure (Gao et al., 2017).

Arsenic is a significant environmental contaminant in several food sources, such as rice, fish, seafood, and domestic drinking water. Elevated levels of arsenic are associated with the development of cancer and other detrimental health effects that impact several organ systems (Naujokas et al., 2013). The gut microbiota of 5-week-old ICR mice exposed to arsenic (3 mg/L drinking water), iron (5 mg/L drinking water), and the combination of both chemicals for 90 days was shown to be modulated (Guo et al., 2014). Furthermore, a recent study has revealed that a significant enrichment of genes is involved in vitamin synthesis within the gut microbiome of individuals with modest exposure to arsenic. This finding implies a potential protective response to As exposure (Chi et al., 2017).

## 5 Conclusion

The gut microbiome plays a critical role in the modulation of immune responses and the maintenance of immune tolerance, which directly or indirectly points out the importance of gut microbiota in the pathogenesis of various diseases. Predisposing factors, including diet, medicines, lifestyle, and environmental influences, are described as having an essential role in the composition of the gut microbiome. It can be said that the effects of these factors are specifically stated as some of the principal determinants of the gut microbiome. A diet low in fiber provides a less diverse population to the gut microbiota, and poor dietary choices such as high sugar and fat may cause microbial imbalance and dysbiosis. On the other hand, physical activity supports the diversity and stability of the microbiome community, while the sedentary lifestyle influences the reduced diversity of the microbiome. Overwhelming stress decreases gut microbiome stability, causing a change in gut motility and permeability in a way that activates dysbiosis. Medications also significantly influence the composition of the gut microbiome. Antibiotics are uniquely powerful because they kill harmful and beneficial bacteria, reducing microbe diversity and potentially even causing dysbiosis outright. Nonetheless, probiotics could help restore the microbial imbalance by reintroducing beneficial microorganisms. Pollution exposes the microbiome to toxins and pollutants. This process leads to the disruption of the microbial communities by favoring the growth of pathogenic bacteria. Therefore, different agents and conditions can lead to changes in the gut microbiome, which can be related to the pathogenesis of various diseases. By understanding these factors, we can get valuable insights into how to intervene to reduce the chances of a disease. However, data about the exact interactions of microbiome changes and disease progression is limited. To this end, further research and clinical trials are needed to unveil how the gut microbiome participates in health and disease.

Future efforts should focus on optimizing microbiome-based therapies and integrating them into personalized medicine approaches to promote human health and more effectively manage disease. Research should be targeted to understand the metabolisms in the gut microbiome change. Through more research, effective interventions can be made in mechanisms involved in the gut microbiome for the treatment of related diseases or modification in the composition of bacteria living in the gut. Also, future research on the gut microbiome promises transformative insights into its role in human health and disease. Advances in microbiome sequencing and personalized medicine could lead to tailored probiotic and prebiotic therapies, optimizing individual health outcomes. Furthermore, interdisciplinary approaches integrating genomics, metabolomics, and immunology will drive innovative interventions.
